# Preserving the owner’s autonomy in networks of patient registries and biobanks

**DOI:** 10.1186/1750-1172-9-S1-P3

**Published:** 2014-11-11

**Authors:** Martin Lablans, Dennis Kadioglu, Marita Muscholl, Frank Ückert

**Affiliations:** 1 University Medical Center, Mainz, Germany

## Background

To achieve statistical significance in rare disease research, bio- or data samples taken from one patient registry or biobank may need to be complemented by those of other institutions [1,2]. While a first overview of potential research partners can be obtained using public catalogues as established by BBMRI [3] or Orphanet [4], this article focuses on mediation services, which provide deeper insight on available material using criteria-based search on fine-grained, non-aggregated datasets. Until now, these datasets were provided either beforehand via regular uploads (central search, e.g. CRIP [5] and the NCI’s specimen resource locator [6]) or on-demand via distributed queries (federated search, e.g. i2b2 SHRINE [7] and EHR4CR [8]). However, both ways give third parties whom the data or sample owners may neither know nor trust insight into their databases.

The requirement for self-disclosure places owners in a dilemma: On the one hand, they want to contribute to promising collaborative research projects. On the other hand, they “frequently hold proprietary views on their data” [9] and want to carefully consider with whom to share their assets collected over years without facing pressure of justification for rejecting a proposal.

## Results

We propose a method to search distributed databases, yet fully keep their owner’s data sovereignty: The *decentral search* exploits distributed, heterogeneous, highly sensitive datasets from equally heterogeneous systems for overarching research questions. Similar to other federated searches, the decentral search detects matching material in distributed data stocks. However, their query mechanism is replaced by a novel request mechanism that involves the owner with a high degree of control, who can (decentrally using their own registry or biobank systems) decide if and what to answer based on a specific project proposal. As no datasets ever leave their institution, they can reject projects without risking their good standing as a cooperative scientist. While the decentral search sacrifices real-time answers, it leads to several beneficial side effects: improved data protection due to data parsimony, tolerance for incomplete data schema mappings, flexibility with regard to patient consents and decreased effort when the network is initially joined.

## Conclusion

The decentral search allows to exploit bio- or data samples while fully preserving their owners’ data sovereignty. It is employed in the Consortium for Translational Cancer Research, one of the six German Centres for Health Research comprised of eleven university hospitals. The decentral search also marks the centrepiece of the *OSSE* national registry of rare diseases.
